# Towards evidence‐based policy in melanoma screening and skin self‐examination education

**DOI:** 10.1111/jdv.70189

**Published:** 2025-12-23

**Authors:** Sinem Ornek Ozdemir, Jason Shourick

**Affiliations:** ^1^ St John's Institute of Dermatology, Guy's and St Thomas' NHS Foundation Trust London UK; ^2^ Department of Clinical Epidemiology and Public Health Toulouse University Hospital Toulouse France

Policies aimed at increasing public awareness of the early signs of melanoma through education on proper skin self‐examination techniques remain an essential strategy for early detection.^1^ Leading dermatological societies emphasize this need and have developed educational materials to promote awareness and encourage regular self‐examinations. In pursuit of this goal, educational programs have long shown their efficacy and, for some policies, their cost‐effectiveness^2^ or implementability.^3^

Spadafora et al.^4^ performed a clinical trial investigating the efficacy of passive education using a booklet explaining the ABCDE and UD rules, compared with active education where the same booklet was explained by a dermatologist, among non‐medical individuals in correctly identifying lesions at risk for melanoma. While both types of interventions have proven some efficacy in the recognition of melanoma, direct comparisons are still lacking. The authors concluded that participants receiving active education were more likely to correctly identify lesions at risk for melanoma, apply diagnostic rules appropriately and retain relevant information over time.

Trials investigating specific educational strategies and their comprehension are a key element of policy development. Two very positive aspects of this study are a consideration for the precise way of presenting educational material as well as which precise rule led participants to the diagnosis. These aspects are rarely considered but are key elements in the evolution of material and its proper usage.

However, while these findings are intriguing, such face‐to‐face education is challenging and costly to implement in clinical practice, where time and resources are limited. Therefore, future investigations of cost‐effectiveness and implementation strategies would be necessary to ensure the proper usability of these results in public health policies.

Another noteworthy aspect of this study is that participants were individuals already attending dermatological skin examinations and presented a high pre‐intervention performance. This suggests individuals with a preliminary awareness or a high‐risk status. However, as noted by Rose in his seminal article on the paradox of prevention,^5^ policies should target all populations, as most avoided cases will come from lower‐risk profiles. Therefore, aiming at reinforcing an already relatively competent individual, even with a high‐risk profile, might not be the best course of action, and this high baseline performance reduces the transferability and generalizability of these findings to the broader public.

Methodologically, the study also raises concerns. The analysis focused solely on whether participants correctly identified lesions at risk for melanoma, reporting a global positive predictive value (PPV) rather than individual participants' diagnostic accuracy. This raised multiple issues. First, PPV is sensitive to baseline prevalence. In this study, the prevalence was artificially set to 0.5, far from the real‐world prevalence of naevi at risk for melanoma, meaning that the real‐world PPV would be much lower than the one reported. Second, PPV alone is insufficient to measure the effectiveness of an educational intervention, as it ignores false negatives. Falsely reassuring results are a key aspect to avoid in screening programs, as they might lead to even later diagnosis. Finally, global PPV ignores observations' non‐independence; indeed, having one participant evaluate 60 lesions is not the same as having 60 participants examining 60 lesions. This greatly underestimates p‐values and does not allow for a clear assessment of how much each intervention improved individual performance. These results should therefore be interpreted with caution.

In conclusion, while individual education is important for those with personal or familial melanoma risk, future studies should focus on population‐wide strategies and include cost‐effectiveness and implementability evaluations. Furthermore, methodological rigor for educational interventions should be equal to the one requested for therapeutic clinical trials, as policy outcomes might have very substantial effects on population health.

## CONFLICT OF INTEREST STATEMENT

Neither of the authors has any conflicts of interest to declare.

## Data Availability

Data sharing not applicable to this article as no datasets were generated or analysed during the current study.
